# Where communities intermingle, diversity grows – The evolution of topics in ecosystem service research

**DOI:** 10.1371/journal.pone.0204749

**Published:** 2018-09-28

**Authors:** Nils Droste, Dalia D’Amato, Jessica J. Goddard

**Affiliations:** 1 Department of Economics, UFZ—Helmholtz Centre for Environmental Research, Leipzig, Germany; 2 Department of Economics, Martin-Luther University Halle-Wittenberg, Halle (Saale), Germany; 3 Department of Development, Environment and Territory, FLACSO—Facultad Latinoamericana de Ciencias Sociales, Quito, Ecuador; 4 Helsinki Institute of Sustainability Science, Department of Forest Sciences, University of Helsinki, Helsinki, Finland; 5 Energy & Resources Group, University of California, Berkeley, California, United States of America; KU Leuven, BELGIUM

## Abstract

We analyze how the content of ecosystem service research has evolved since the early 1990s. Conducting a computational bibliometric content analysis we process a corpus of 14,118 peer-reviewed scientific article abstracts on ecosystem services (ES) from Web of Science records. To provide a comprehensive content analysis of ES research literature, we employ a latent Dirichlet allocation algorithm. For three different time periods (1990–2000, 2001–2010, 2011–2016), we derive nine main ES topics arising from content analysis and elaborate on how they are related over time. The results show that natural science-based ES research analyzes oceanic, freshwater, agricultural, forest, and soil ecosystems. Pollination and land cover emerge as traceable standalone topics around 2001. Social science ES literature demonstrates a reflexive and critical lens on the role of ES research and includes critiques of market-oriented perspectives. The area where social and natural science converge most is about land use systems such as agriculture. Overall, we provide evidence of the strong natural science foundation, the highly interdisciplinary nature of ES research, and a shift in social ES research towards integrated assessments and governance approaches. Furthermore, we discuss potential reasons for observable topic developments.

## Introduction

The ecosystem services (ES) concept was developed in the 1970s and 1980s by conservation biologists and ecological economists to encourage decision-makers to recognize and attend to socio-ecological linkages [1–2]. The field has grown exponentially since the late 1990s [3–6]. The concept was quickly adopted as a research frontier and boundary object for evaluating social-ecological systems and as a basis for managing environmental change [3,7–8]. Generally, ecosystem services describe contributions from ecosystems to human well-being, and they have been categorized into ‘provisioning’, ‘regulation and maintenance’, and ‘cultural’ services [9]. The concept thus covers topics as wide-ranging as agricultural production (a provisioning service), control of erosion rates (a regulating service), and amenities of a cultivated landscape (a cultural service), to name just a few.

The ES concept thus encompasses and bridges several disciplines under the umbrella of sustainability science. Research stemming from the natural and social sciences to the humanities contribute to a myriad of interpretations and applications of the ES concept [6,10–11]. Previous reviews indicate that the ES literature remains dominated by ecology and economics, but that the research is growing increasingly diverse and multidisciplinary [3,5]. The concept has furthermore been actively promoted at science-policy interfaces [12–14] and in grey literature supporting research and practice outside of academia [15–17].

The highly interdisciplinary and socially constructed nature of the ES framework invites a series of ontological and epistemological challenges [18–19]. In this regard, the most relevant ES research is today largely occupied with conceptual and methodological development, refining definitions, and applications and tools for ES mapping, valuation and policy implementation, especially in light of equity, development and market issues [20–26]. One of the main areas of discussion on ES research has focused on theoretical and practical implications and limitations of the concept, especially around a neo-liberal (utilitarian) framing supporting the appropriation of nature, monetary valuation and the potential danger of commodifying ES [27–31]. Partially in response to these critiques, several authors have identified gaps and potential avenues for ES research. For example, La Notte et al. [32] pointed out that the traditional research approach using the ecosystem services cascade framework emphasizes the end-use benefits of ecosystem services, while more emphasis on the underlying complexity of ecological systems would be beneficial. Reyers et al. [33] highlighted this complexity as a challenge to the ES concept and argued for ES research to engage more with social-ecological systems frameworks, which include coupled social-ecological processes, ES interactions, human wellbeing, and non-linear feedback loops. Lele [19] and Dempsey and Robertson [28] called for the contribution of political ecology, environmental sociology, ecological anthropology and human geography, to challenge the dominant influence of economists in the current literature. Abson et al.’s [3] review of the sustainability vernacular within academic ES research identified an emphasis on descriptive (as opposed to normative or transformative) research. Chaudhary et al. [5] highlighted emerging opportunities for the interdisciplinary ES research to engage more with topics such as poverty and justice. Consequently, there are many perspectives on what ES research is or should be about. The historical evolution of ecosystem services research is complex and requires new methods to capture the concept’s many uses across disciplines and the rapidly evolving trends and themes in ES research.

This methodological challenge provides the impetus for our analysis, which aims to capture the internal diversity of ES research over time. Our research question is: *What are the main topics in ES research and how have the topics changed over the last 26 years time*? Previous studies have only evaluated small subsets of the ES literature (a few hundreds of articles), often based on highly cited literature [3,5,6,22,34–35]. This study, instead, explores the entire Web of Science literature on ES (over 14 thousand articles). Given the magnitude of the scientific literature dealing with the topic, we employ an unsupervised machine learning algorithm, latent Dirichlet allocation (LDA), which allows us to process an unprecedented volume of ES research articles [36] (see for a notable exception in sustainability research [37]). Deriving clusters of documents belonging to topics through an occurrence probability matrix of words in documents, we analyze the literature without predefined topics. Both the most representative terms for each of the topics and the topics’ relative size are generated from the literature corpus across three time periods between 1990–2016. The quantitative approach adds methodological rigor to the process of capturing content and developing topic clusters is reproducible, and other researchers can explore the data interactively (http://nils.droste.io/2017/10/05/ES_LDA/) and find the analytical code at our github repository (https://github.com/NilsDroste/ES-LDA). Based on the LDA-determined topic areas, we interpret the results to describe 1) the relative importance in the overall ES narrative based on their share of the overall corpus for each period and 2) the link of topics within the ES literature across periods. We aim to provide both a descriptive picture and highlight the linkages between different types of topics within ES research in order to clarify the topics around which different research communities have evolved. This enables us to capture some of the “multiple understandings […] of human-nature relationships” in ES research [18]. Some dynamics of thematic composition are displayed through comparisons across periods.

The structure of the article is the following. We present related literature in section 2, expound our methodology in section 3, provide the results in section 4, and discuss topic developments in section 5. In section 6, we conclude briefly.

## Debates within ES literature

Several reviews on ES exist, and we employ several of their insights to structure our analyses and relate our results to their findings. Gómez-Baggethun et al. [6] articulated four stages of ES research, practice, and economic theory: utilitarian framing (1960s - 1990s), monetization (begun 1960s - accelerating in 1990s), appropriation, and exchange (both began in the 1970s - accelerating in 2000s). They find that the utilitarian framing of the initially rather metaphorically used ES concept could open up the way for “market logics in the field of nature conservation” (pp. 1215 in [6]). Dempsey and Robertson (pp. 773 in [28]) furthermore point out that tensions between rather neoliberal doctrines and “other elements of global development strategy” can be found in ES research, and that these create entry points for critical scholars who aim to engage and change the discourse. Similarly, Raymond et al. [8] argue that while a focus on direct use and economic quantification is sometimes appropriate, a more diverse set of metaphors for human–environment relationships broadens the scope and would allow for a better deliberation between perspectives.

Abson et al. [3] summarised the state of ES research and examined its contribution to sustainability knowledge based on a review of 1,388 top cited ES scientific articles. They found nine main discourse topics in the literature: valuation, conservation, management, carbon, diversity, water, pollination, forests, biomass. Their analysis focuses on the extent to which ES research develops knowledge for sustainability goals. They categorized each paper’s descriptive, normative, and transformative focus. They identified that most research in their sample is descriptive in nature, rarely explicit in its normative position, and under-developed with respect to transformative knowledge essential to sustainability. This is perhaps related to findings from Seppelt et al. [38]. They aimed to quantitatively review the methods and approaches found in 153 regional case studies of ecosystem services between 1980–2010 and identified a lack of methodological consistency across studies. Similarly, Naeem et al. [29] argue for ‘getting the science right’ in payments for ecosystem service projects and propose principles of scientific integrity such as defining a baseline, monitoring along consistent metrics, and a recognition of ecosystem dynamics.

Chaudhary et al. [5] focused their review on the evolution of ES research through time with a quantitative analysis of ES research disciplines coupled with a qualitative assessment of “discursive-institutional spirals” over time. They grouped five periods according to important events and identified each period's main actors, institutions, and disciplines (pre-1997: early academic conceptions, 1997–2000: expanding interest, 2001–2004: uptake by global actors, 2005–2009: global reporting, 2010–2013: IPBES institutionalization). While they conclude that both ecology and economics are the most important disciplines they also identified that boundary organizations played a critical role in the institutionalization of the ES discourse. Both Abson et al. [3] and Chaudhary et al. [5] found that ES research is increasingly represented across social and natural science disciplines, but that research topics remain relatively siloed. In a recent review, van den Belt and Stevens [35] characterize value frameworks for ES and find that a discourse has evolved across utilitarian and intrinsic value frames in the most cited literature of first four years of the Ecosystem Services Journal (2012–2015).

Furthermore, there is an ongoing conceptual development in ES research. Chan et al. [39] broached the concept of relational values in human-nature relationships in an attempt to expand the long-standing debate pitting instrumental and intrinsic values against one another in ES. Correspondingly, Jacobs et al. [40] show the diversity of valuation methods and call for an integrative valuation practice that includes diverse values. At the science-policy interface, the Intergovernmental Science-Policy Platform on Biodiversity and Ecosystem Services (IPBES) community produced a conceptual framework of ‘nature’s contributions to people’ that incorporates ‘diverse conceptualizations of multiple values of nature and its benefits‘ [41]. The IPBES framework incorporates and articulates various sources and concepts of values regarding nature’s contribution to people and society such as anthropocentric values like instrumental, socio-ecological relational values, and ecocentric intrinsic values [12, 41–42]. Calls to assess ES with regard to the underlying complexity of socio-ecological systems and principles of sustainability, and the breadth of the IPBES values guide suggest a research and science-policy agenda that strives towards more inclusive and holistic understanding of the role and potential for ES work [40, 43]. We therefore set out to capture past and current states of thematic diversity within ES research and display some dynamic evolution of topics that different research communities analyze.

## Methods

### Data collection

We conducted a bibliometric analysis of the scientific literature dealing with the concept of ES. We retrieved Web of Science (WoS) core collection (all years, by topic) data using the string “ecosystem service*” (https://clarivate.com/products/web-of-science/). The search resulted in over 15,000 records. These records include authors’ names and affiliations, title, abstract, full record and cited references but no full texts.

In order to outline the evolution of the ES literature over time, we downloaded the WoS entries for three periods 1990–2000, 2001–2010, and 2011–2016. This choice corresponds broadly to the phases of ES research development from Chaudhary et al. [5]: 1990–2000: early research, 2001–2010: global uptake, and 2011–2016: institutionalization (see section 2). From the original data set we removed double entries and those records that did not provide an abstract. This collection of records represented the corpus of text for our analysis (Table 1).

**Table 1:** Dataset record counts from initial pull and final dataset by time period.

Years	Records available	Final dataset
1990–2000	136	108
2001–2010	2,719	2,521
2011–2016	12,183	11,489
All years	15,038	14,118

The final data set excludes records with empty abstracts and double entries. *Source*: Author’s representation based on WoS.

### Content analysis

The following analyses were performed within the **R** Environment [44]. The respective code can be found on a public github repository at https://github.com/NilsDroste/ES-LDA.

***Descriptive statistics*** include the geographical origin of the articles and the most frequent authors’ keywords. The geographical provenience of the articles is evaluated based on the authors’ affiliations and then represented graphically on a map. It is important to note that the map shows where the articles are written, and it is not indicative of the geographical location of data or study sites. It should be noted that the number of keywords attributed by the authors may vary across documents, from 1 to 6.

***The computational content analysis*** of the abstracts, representing the main analysis, was performed using the latent Dirichlet allocation (LDA) method [36,45]. The software implementation was provided by the ***lda*** package [46], ***tm*** [47], and ***SnowballC*** [48]. For visualizations we used ***LDAvis*** [49], ***ggplot2*** [50], ***rworldmap*** [51], and the ***sankey*** [52]. The overall text processing procedure is an adaptation of the **R** source code provide by Knutas et al. [53]. The abstracts required specific preprocessing for analysis [47–48]. Spaces, punctuation, acronyms, numbers and symbols were removed. Words were stemmed, meaning they are reduced to their underlying root form, and terms which occur fewer than 5 times have been removed. This resulted in a “bag” of words for each of the abstracts retrieved [54–55]. The most salient terms were identified based on overall word frequency in the abstracts. The LDA method has been developed in computational sciences as a form of natural language processing. The underlying assumption is that text documents are composed of several unobserved topics, and these can be revealed based on the likelihood of word co-occurrence. Basically, in LDA each document can semantically be described by its mixture of latent topics, where each topic is a dirichlet distribution over words and where words may thus occur in the distribution of several topics [36]. Every document consists of a particular set of words. The probability that word *w*_*i*_ is contained in document *d*_*k*_ is given by

$$P(w_{i}|d_{k})=\sum_{z=1}^{Z}P(w_{i}|z)P(z|d_{k})$$

where *z* is the latent topic, *P*(*w*_*i*_/*z*) is the probability of word given topic *z*, and *P*(*z*|*d*_*k*_) is the probability of topic *z* given the document *d*_*k*_ [36,56].

In the unsupervised process of learning the set of topics LDA employs a multinomial dirichlet distribution and infers a posterior distribution through a variational Bayes approximation [36]. Every document is being repeatedly assigned a topic. The posterior topic distribution *P*(*z*|*d*_*k*_) provides probabilities for topic assignments of each document. From there it is possible to identify the most probable topic for each document and the most probable documents for each topic. The number *Z*, topics to be identified, must be determined a priori. Topic number selection can be optimized in terms different measures, such as accuracy [57], density [58], latent concept modeling [59] or Bayesian Markov chain Monte Carlo algorithm [60]. As all of these different optimizations computed through the ***ldatuning*** package provided by [61] resulted in over 400 topics for the last period, we have chosen to limit topics to an interpretable number which we hold constant over time in order to ensure comparability across periods and display (dis-)continuities in the development of topics over time. This required us to choose a sufficient, but not excessive number of topics to reveal the literature’s internal variability without hampering its interpretation with information overload for the sake of model accuracy. We chose the total number of topics in an iterative and literature driven process. First, we set the model to extrapolate six topics, which is the default option proposed by Knutas et al. [53] whose code we adapted. Based on the results, we chose to increase the number of topics to nine to capture more subtlety across topics in the literature. Our choice of topic numbers is in line with the number of topics outlined in a content analysis of the most cited literature on ES by Abson et al. [3]. This way, we can provide some comparability across reviews.

The results of the LDA analysis can be explored interactively with a web-based visualization (http://nils.droste.io/2017/10/05/ES_LDA/). In the interactive plots, the key term frequencies are represented in a histogram, while the topics are positioned on a principle component plot based on their semantic relationship. The topics are represented as circles, where circle size measures the relative topic share. The browser-based display provides the option to identify the key terms that are most relevant for each topic. The relevance of a word *w*_*i*_ for a topic *z* is defined as *λ* × *P*(*w*_*i*_|*z*) + (1 − *λ*) × *P*(*w*_*i*_|*z*)/*P* (*w*_*i*_) for values of 0 ≤ λ ≤ 1 [49]. A lower λ corresponds to more exclusively relevant key terms, while higher values of λ retain more frequent key terms. Topic descriptions (see section 4.2) were derived from LDA generated salient key terms at different λ and abstracts of the top 20 most probable articles for each topic (see S1–S3 Tables and S1–S3 Visualizations).

***The interpretative derivation of topic development and linkages over time***. We took an iterative and collaborative approach to interpretation of topic development over time. Firstly, we conducted an analysis of LDA results at various λ values and the top 20 papers contributing to topic clusters to derive content and methods. In section 4.2, we provide a description of the topics content. In order to map the evolution of the topics in time, we compared the topics found across the three time periods according to representative key terms occurring in several topics. Secondly, we tracked topic development. In order to enhance transparency, we provided a list of key terms that we used to identify and track topic development in section 4.3. Through discussions we refined both the labeling and the linkages, and thus the graphical illustration of how and where similar content can be found in another period’s LDA derived topics. The list of the top 20 most probable papers for each of the topics across all three periods available in the supporting information (S1–S3 Tables) and an interactive LDA visualization through either S1–S3 Visualizations (see instructions in S1 Text) or online at http://nils.droste.io/2017/10/05/ES_LDA/.

## Results

### Descriptive statistics plots

**Fig 1:**
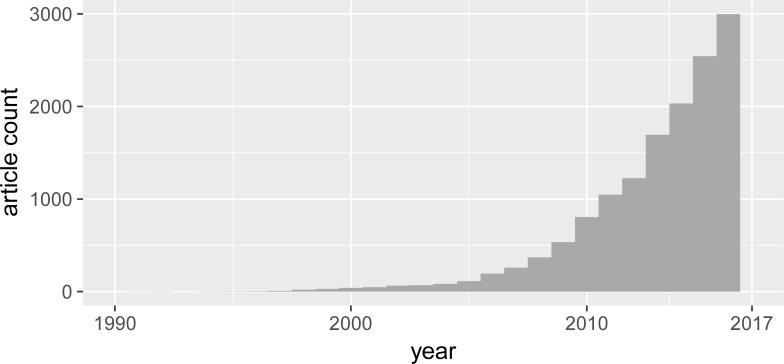
The growth in ES research. *Source*: authors’ representation based on WOS data.

The descriptive statistics plots in this section are based on an analysis of the entire data set. Fig 1 shows the exponential growth in ES literature.

**Fig 2:**
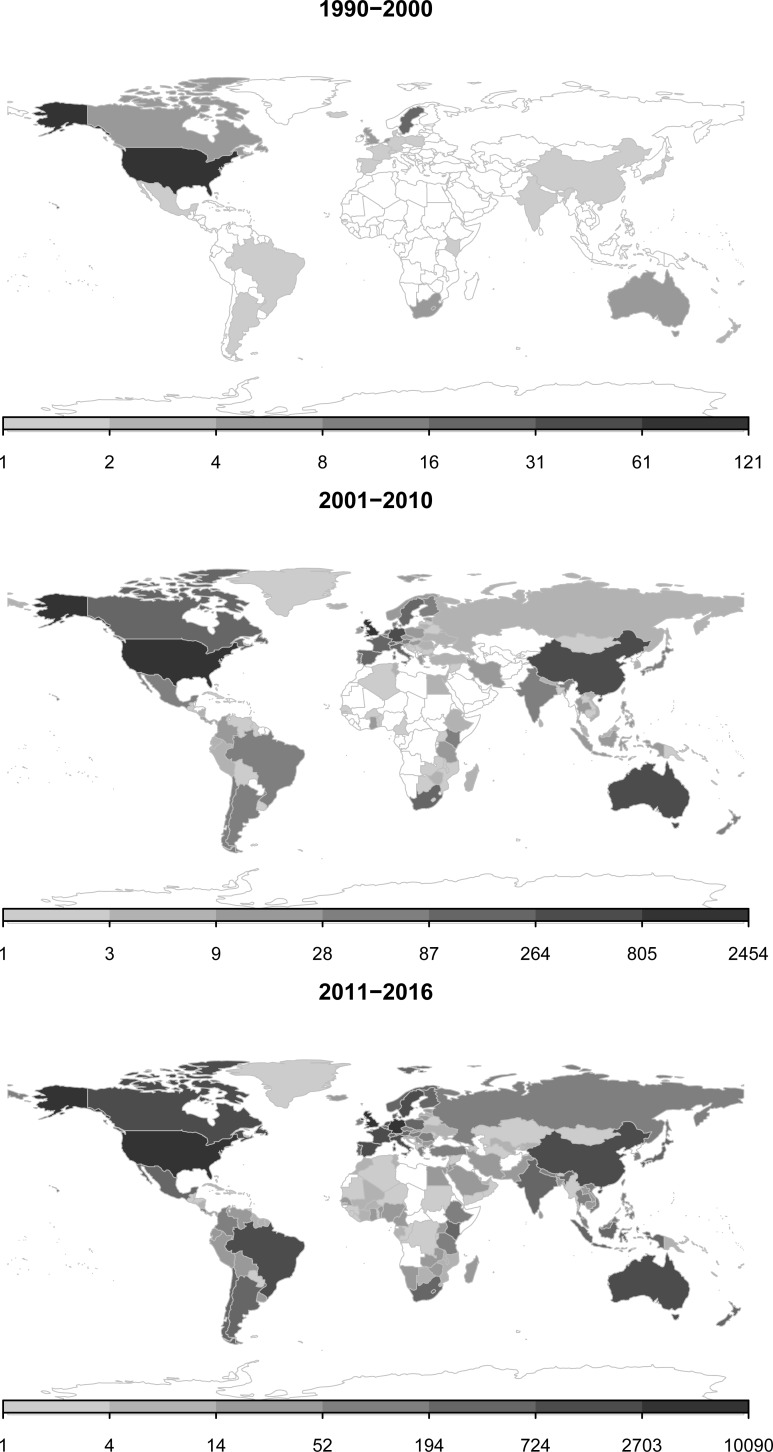
The geographical distribution of ES research. Colour scales represent the count of author affiliation locations. Note the different fixed width logarithmic colour scale breaks. *Source*: authors’ own representation based on WoS.

Fig 2 displays that the early ES research originated mainly from OECD countries. During later periods, the ES research dispersed globally such that in the 2011–2016 period there is a much more equally distributed location of authors home institutions—with a gap in ES research authorship remaining in some African countries.

**Fig 3:**
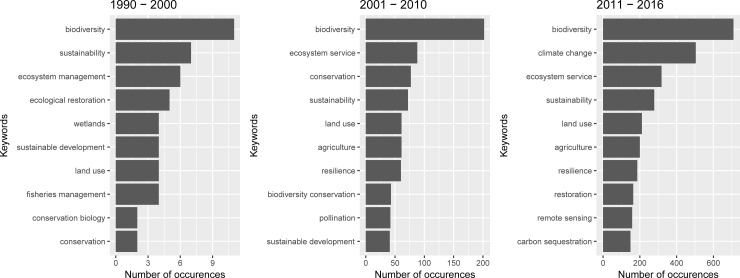
Top author keywords for the research corpi in three periods. Note the different x-axis-scale of the plots for each period. *Source*: authors’ own representation based on WoS data.

Fig 3 shows the top 10 author supplied keywords and thus general content trends. The graph shows that biodiversity is essential to ES research. While “sustainability” was ranked second during the first period, “ecosystem service” and “conservation”, and “climate change” and “ecosystem service” became more frequent keywords in the second and third periods, respectively. “Resilience” and “agriculture” appear from the second period onwards. “Remote sensing” only becomes prominent during the 2011–2016 period.

### Content analysis

**Fig 4:**
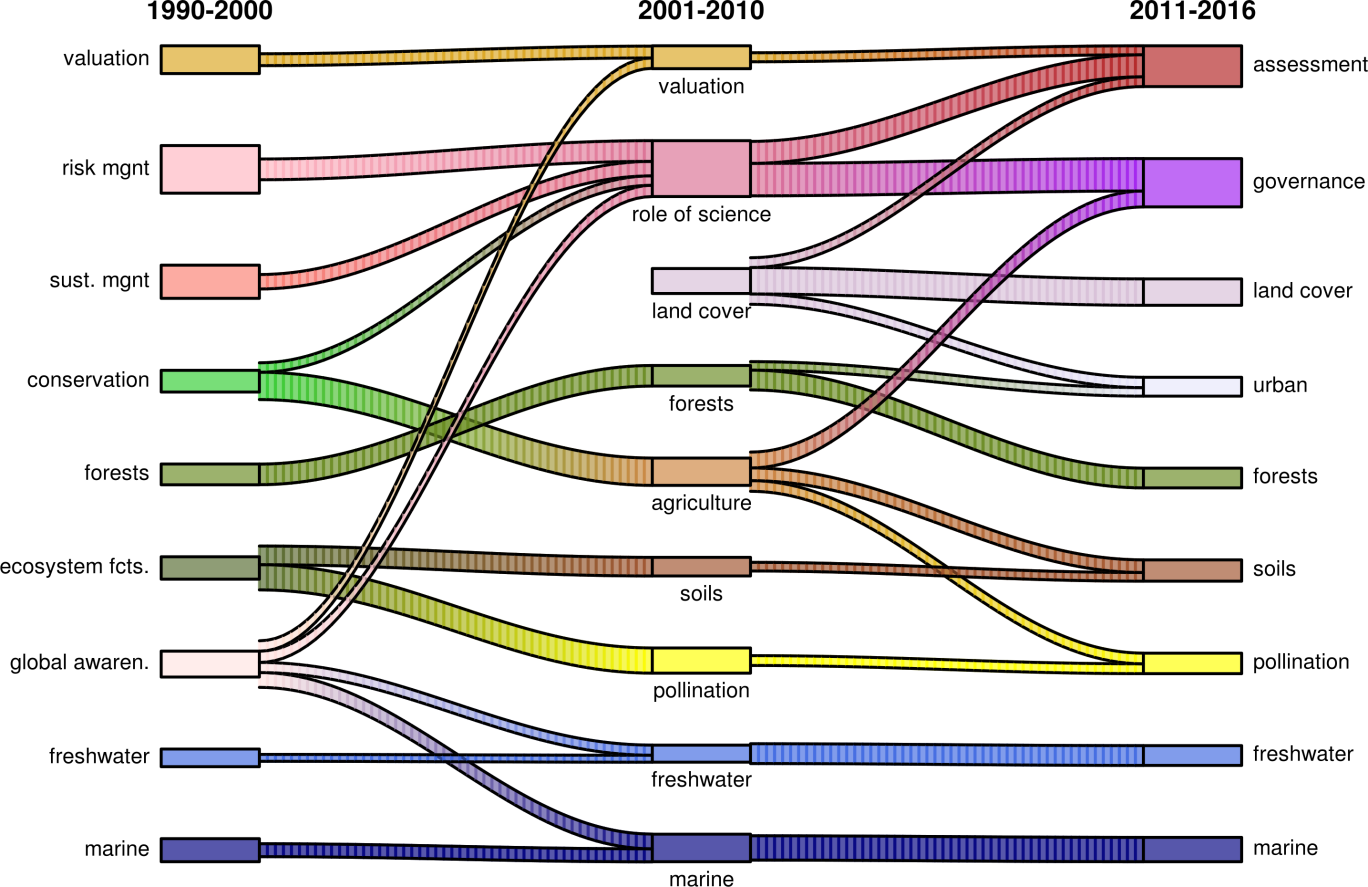
The development of ES topic clusters over time. The height of the topic boxes represents the relative topic proportion within each period. Note that the number of assessed articles increases over time (1990–2000: N = 108, 2001–2010: N = 2,521, and 2011–2016: N = 11,489). The links between periods have interpretatively been deducted from LDA results. There is no particular order of topics except for clarity of linkage exposition. *Source*: authors’ elaboration based on WoS data.

For each of the periods, the LDA algorithm provides nine topics with a probability distribution over words. Here, we describe the topics in more generic terms. A graphical overview of the topics for each period, their relative size, and their development can be found in Fig 4.

References to the 20 most representative, as in most probable, articles for each of the topics and a table of the assigned topics for all articles per period can be found in the supporting information (S1–S3 Tables). The online interactive results provide far greater detail (http://nils.droste.io/2017/10/05/ES_LDA/). For each of the topics we elaborate on what kind of ecosystems are assessed, and, as far as possible, what kind of ES are addressed. In Table 2 we describe the topics emerging from the analysis in alphabetical order and their primary methods, which were largely derived from the 20 most probable articles per topic (see S1–S3 Tables). Topic linkages between periods are presented in section 4.3.

**Table 2:** Topics in ecosystem service research.

Topic	Thematic focus	Methods
Agriculture	Food production, provisioning services, conservation, payments for ecosystem services, carbon sequestration, policy measures	Economic valuation (willingness to pay / accept), science and technology studies, trade-off analysis, policy evaluation, scenario methods
Assessment	Ecosystem services assessments, science-policy interfaces of national ecosystem service assessments and planning procedures	Mapping techniques (spatially explicit) ecosystem service flow models, participatory (scenario) planning, Bayesian belief networks,
Conservation	Biodiversity loss, (regional) environmental change, climate change, agricultural landscapes, agroforestry, management strategies	Impact / effectiveness studies, (financing) strategy evaluations, driver and impact analyses, spatio-temporal and functional assessments
Ecosystem functions	Ecosystem composition and fragmentation, species communities and richness, terrestrial ecosystems above and below ground,	Experiments, field study sampling, meta studies, species distribution studies, functional assessments, process and (material) flow analyses
Forests	Management practices, land use / cover change, species composition, forest fires, carbon storage, urban green space, alien species	Disturbance recovery studies, impact evaluations, land cover assessments, longitudinal studies, satellite imagery
Freshwater	Hydrology, retention services, flow regimes, sediment streams, wetland and marsh management, material discharge and uptake, hydropower	Hydrological models, GIS analyses, physical, chemical and biological analyses, blue, green, gray water assessments, morphological studies
Global awareness	Total economic value, nitrogen retention and drainage, (sea)food production, reefs, human demand, ecological thresholds, natural capital	Economic valuation, GIS analyses, large scale biophysical-monetary assessments, quantitative evaluations,
Governance	Conservation, management, sustainable development, policies, research, social change, challenges, adaptation, environment	Network analyses, legitimacy and accountability studies, system analyses, transdisciplinary research, policy evaluation
Land cover	Land use change / cover models and scenarios, sensors, surrogates, accuracy, resolution, spatial data, classification, patterns	Satellite imagery and remote sensing, spatially explicit analyses, geo-spatial assessments, maps, simulations, lidar
Marine	Habitats, coral reefs, mangroves, fishery, climate change, functional diversity, species composition, extinctions	Functional assessments, genetic studies, species distribution analyses, diversity assessments, global change analyses
Pollination	Supporting services, inter-insect relations, predators, biological and chemical pest-control, habitat structure, plant communities	Field samples, management intensity and type studies, crop / harvest assessments, diversity / composition evaluations
Risk management	Ecological processes, ecosystem services, stressors, man-made disturbance, ecological resilience, response management	Spatio-temporal ecosystem models, risk assessments, toxicity tests, management studies, system-wide driver-impact evaluations
Role of science	Research, policy, sustainability, frameworks, social change, systemic analyses, knowledge, practitioners, communication, civic engagement	Transdisciplinary research, socio-ecological models, qualitative studies, knowledge classifications, conceptual studies
Soils	Soil structure, species community, functional diversity, carbon storage, crop production, bioenergy, feedstock, micro-biota, fertilizer	Bio-geo-chemical analyses, diversity assessments, functional assessments, cropping system analyses, land management studies
Sustainability management	Development, restoration, humans, socio-ecological systems, capital, planetary perspectives, future, life support, paradigms	Conceptual studies, system boundary assessments, history to future extrapolations, capital accounting, response formulation
Urban	Human livelihood, urban sprawls, green spaces, parks, recreation, health, planning, economic value, rural dwellings, households, tourism	Land cover assessments, GIS analyses, micro-climate models, economic valuation, urban planning, surveys, cultural evaluations
Valuation	Methods, (non-)market goods and services, value concepts, aggregation, discounting, accounting, benefits, costs	Revealed preference methods, contingent valuation methods, cost-benefits analyses, total economic value aggregation, surveys

Source: Author’s elaboration based on from Latent Dirichlet Allocation analysis results of WoS data.

### Topic development

We used the results of the LDA analyses from each period to assess the content of each topic descriptively. We then tracked topic development through collaborative and iterative qualitative analyses of the content (see section 3.2). In order to provide transparency about the corresponding qualitative process, Table 3 provides an overview of key terms that we used to track linkages between topic across periods. In combination with the interactive visualizations (http://nils.droste.io/2017/10/05/ES_LDA/) the table provides a certain degree of reproducibility.

**Table 3:** Topic linkages and the key terms used for tracking.

Linkages			Shared stemmed key terms across different λ
Agriculture	→	Governance	agricultur, agrobiodivers, biodivers, benefit, conserv, develop econom, ecosystem, environment, incent, landhold, manag, market, payment, pes, polici, rancher, resourc, servic, sustain, system
	→	Pollination	agricultur, biodivers, increas, farm, manag, provid, servic
	→	Soils	agricultur, bionergi, carbon, ecosystem, emiss, energi, farm, ghg, land, product,
Conservation	→	Agriculture	agricultur, biodivers, conserv, ecosystem, land, polici
	→	Role of science	biodivers, chang, conserv, ecosystem, natur, process, polici
Ecosystem functions	→	Pollination	abund, comuniti, plant, pollin, rich, speci
	→	Soils	biomass, function, grassland, plant
Forests	→	Urban	area, citi, develop, ecosystem, green, landscap, natur, park, protect, urban, space
Global awareness	→	Freshwater	freshwat, wetland
	→	Marine	coral, fish, global, marin, popul, ocean
	→	Role of science	conserv, ecosystem, function, human, natur, servic
	→	Valuation	account, costanza, estim, reserv, servic, valu, yuan
Land cover	→	Assessment	approach, assess, base, ecosystem, indic, inform, method, model, spatial, studi,
	→	Urban	area, ecosystem, result, servic, studi,
Risk management	→	Role of science	address, develop, ecolog, ecosystem, human, integr, manag, problem, process, scientist, servic, societi, system
Role of science	→	Assessment	approach, ecolog, ecosystem, framework, integr, provid, servic, system
	→	Governance	approach, biodivers, challeng, conserv, develop, ecolog, ecosystem, environment, human, knowledg, local, manag, natur, neoliber, polici, research, resourc, scienc, servic, social, sustain, system
Sustainability management	→	Role of science	develop, ecolog, manag, natur, servic, sustain, system
Valuation	→	Assessment	assess, base, benefit, ecolog, ecosystem, emergi, environment, evalu, method, provid, servic, studi, valu, valuat,

Source: Author’s elaboration based on LDA analysis of WoS data.

In the first period, from 1990–2000, five out of nine topics mainly deal with ecology and land use (conservation, forests, ecosystem functioning, freshwater, and marine) while four topics at address social issues and practices (valuation, global awareness, risk management, and sustainability management). A core topic in this time period is dedicated to larger-scale societal impacts on ecosystems and economies, which we thus labeled global awareness. Concepts in the global awareness topic branch into different topics in later periods, such as valuation, the role of science, and freshwater ecosystems. We furthermore find that the stream of research with a focus on sustainability, risk management and global awareness precedes the reflective role of science for sustainability transformations topic. The second period (2001 to 2010) shows an increase in the share of natural science-related topics, with foci on forests, soils, pollination, freshwater and marine ecosystems. Pollination occurs as an individual topic in the second period and becomes an important (policy) issue globally, as reflected by the recent assessment by Intergovernmental Science-Policy Platform on Biodiversity and Ecosystem Services [62]. Land cover and agriculture research areas address topics at the interface of land use practices and ecosystem functioning. Land cover links to the urban ES topic that emerges in the 2011–2016 period for their commonality in using spatial data. The agriculture topic links with governance approaches, soil research and pollination and thus submerges into various topic clusters that then include agricultural issues. Valuation topics continues in the second period with more detailed, often case-study economic analyses. The role of science topic relates to ES governance research and integrated assessments in the 2011–2016 period. Furthermore, the ratio between natural and social science-related topics remains constant compared to the preceding period, but the composition changes. In 2011–2016 there are five natural science topics (forests, soils, pollination, freshwater, and marine), one social science topic (governance), and three topics at an interdisciplinary interface focusing on human impacts on ecosystems and benefits humans may derive from nature’s contributions: assessments (including biophysical and societal valuations), land cover, and urban analyses.

## Discussion

This article is based on a reproducible quantitative analysis of the abstracts of ES literature available in the Web of Science, from which we qualitatively evaluate the evolution of research topics over time. We aimed to capture and display the internal, thematic diversity of ES research through an innovative method, and thus we focus our discussion on both the content and development of topics in ES research (section 5.1) and the value and limitations of the LDA method (section 5.2).

### Content related observations and potential explications

We find that most of the topics emerging from our LDA analysis of ES literature since 1990 align with the topics identified by Abson et al. [3] and Chaudhary et al. [5] despite some differences in framing and labeling. For example, Abson et al. [3] identified nine research topics, including valuation, conservation, management, carbon, diversity, pollination, forests and biomass. However, from a quantitative perspective, we found that the topics’ relative share in the overall literature (i.e. a corpus of 14,118 papers) diverges from the topics’ share found among the most relevant (i.e. highly cited) articles analyzed by Abson et al. [3]. According to the authors, valuation is by far the largest topic (N = 606), followed by conservation (N = 232) and then management (N = 140). Their analysis indicates a heavy social science orientation within the ES research community. Our findings about the relation of social and natural science-based research contradict previous reviews’ claims that the ES concept tends towards monetization and commodification [30–31]. While the most influential literature could be more oriented towards market-based thinking or economic valuation [63–64], such an argument cannot be supported from a quantitative evaluation of the overall ES literature. Rather, we find a substantial and continuous production of predominantly natural science based ES research with a focus on ecological processes and functions with little trace of monetary values or other corresponding key terms within the content analysis. Additionally, our analysis demonstrates that even within the social science ecosystem services topics, some critical perspectives on ES valuation emerge. For example, “neoliber”, “commodif”, or “moral” are among the topic-specific key terms of the governance topic in the last 2011–2016 period at lower λ (more topic specific). That is to say, while there is a substantial amount of social science research on ES which analyze market based instruments, in particular on agriculture, there are also rather critical perspectives that are observable through LDA. This suggests that critical research on ES valuation plays a role in shaping the social science perspective.

With regard to the topic development over time, we structured periods broadly among the phases identified by Chaudhary et al. [5]: 1990–2000: early research, 2001–2010: global uptake, and 2011–2016: institutionalization (see section 3). In the first period, the conservation topic and the global awareness topic serve as hubs or starting points for topics in subsequent periods–which is most likely a response to a growing recognition of (large scale) environmental problems of modern modes of production and a corresponding need for nature conservation. Global awareness spreads into both social science (valuation and role of science) and natural science (marine and freshwater) topics. Conservation feeds into the role of science and agriculture. During the second phase, there is more differentiated research focusing on particular ecosystems and functions, which is more or less in line with themes identified by Chaudhary et al. [5]: e.g. agriculture, freshwater, forestry, or marine. The soil, pollination, land cover, and the role of science topics were not explicitly observed by Chaudhary et al. [5], but this may be due to labeling differences. We find the emergence of the role of science topic particularly interesting because it suggests an actively engaging social ES science community that contributes a reflective research perspective on what effects ES research may and should have. Potentially, the development of such a self-reflective social ES science was a response to early criticism [65–66] and a need for clarification of the boundaries of market based conservation instruments [67]. Nevertheless, through the converging co-evolution of both natural and social science, ES research became much more interdisciplinary and policy-oriented throughout the topic periods. During the last time period, we observe two interesting shifts in the ES research foci. Firstly, the role of science topic and the valuation topic feed into the assessments topic, which is socio-ecological, comprehensive and transdisciplinary. This is in line with the literature: e.g. Jacobs et al. [40] call for an integration of diverse values of nature’s contributions in land use decisions. Secondly, the role of science topic also feeds into the governance topic, which includes institution building for integrative ecosystem (service) management. Calls for a focus on institution building are also reflected in the literature [43,68]. In our view, these shifts could be a result of the growing recognition of the multitude of values that people assign to nature beyond instrumental or even monetary values, especially in science policy interfaces such as IPBES [42].

Our analysis corresponds broadly to observations already made, suggesting the potential for LDA to complement other review-efforts as research publications grow and the ES topic continues to expand across disciplines. We contribute two important insights that are supported by quantitative evidence through our LDA analysis. We observe:

1. a substantial natural science foundation of ES research with a high volume of publications as well as a highly interdisciplinary ES community with important contributions from social science. However, we do not find evidence that there is a major shift towards market based instruments in the greater ES literature corpus. We also note some critical and self-reflective perspectives among the social ES science community, which perhaps contributed to the waning persistence of monetary valuation research for ES;
2. a convergence of several disciplinary perspectives (including economic valuation and land use topics) into an integrated assessment topic that combines various approaches and (e-)valuation methods. Similarly, several topics (including agricultural and role of science topics) influence a broad governance topic that includes analyses of market-based, governmental and bottom-up participatory approaches for ES management.

We would thus argue that the combined contributions of natural and social science make ES research a highly interdisciplinary field which relies on cross-fertilization and a corresponds to an evolutionary change in topics among research communities.

### Methodological considerations

The main value added of the LDA technique is that key terms and topics within a literature’s entire corpus emerge based on the data itself rather than the a priori postulations formulated by researcher(s) through their analytical processes. The unsupervised learning algorithm clusters documents among topics based on their conditional distribution of words. This allows the data to “speak” for itself through conditional word occurrence probabilities. Our own contribution beyond applying the method to ES research has been to track topic development over time though interpretative linkages while holding the number of topics constant. To our knowledge, this is the first fully reproducible unsupervised machine learning algorithm analysis on ES research and link this to an interpretive time series analysis.

Previous review papers summarizing the state or evolution of ecosystem service literature have focused primarily on either top cited publications, e.g. papers with > 15 publications [5] or papers cited at least one citation per year [3] or top cited publications within the *Ecosystem Services* journal [35]. The value of this approach is its focus on the academic productivity of a given journal or paper. It’s ability to capture the full landscape of knowledge production and academic activity, however, is limited. By including the expansive corpus of literature on ecosystem services, our approach does not bias topic development with influential articles in terms of citation count. While there may be drawbacks to leaving out academic influence (such as limitations on the ability to demonstrate which topics are *most influential*), we are able to evaluate the entire spectrum of ES research up to date and evaluate the proportion of contributions from various topics to the evolution of ES research over time. This provides a snap-shot of what most academic ES research output looks like over time. With LDA over an entire corpus of literature, we can effectively answer the question: on what topics are ES researchers spending time, money, and intellectual dedication?

There are limits to our strategy. Our data only includes scientific literature published in English, thus excluding, for instance, grey literature and policy documents, or publications written in languages other than English. Furthermore, we were unable to perform any preliminary screening of the collected articles given the size of the dataset to verify for relevance and adherence with the ES concept. It is thus possible that an undefined portion of the literature mentions “ecosystem service(s)” as a buzzword or post-hoc justification for research, as suggested by Abson et al. [3]. Furthermore, while the choice of nine topics across all periods allowed us to compare our results with topics derived in former analyses [3], and thus helped to substantiate our interpretation, a different choice of topic numbers would most likely result in a somewhat different bag of topics. We chose to opt for inter-publication comparability.

The linkages across periods were developed through interpretative analysis by all three authors. While the LDA analysis of topics for each period is fully reproducible, the interpretation of how these topics link and develop over time may not be, although we provided a list of key terms that we used to track topic development for transparency.

A next step in topic modeling regarding ES research could be the application of dynamic topic models and the influence model [69], which allows for tracking the development and content of a singular topic over time algorithmically, instead of the interpretation and linkage creation conducted by the authors. Another step could be the application of hierarchical topic models which allows for topics to be correlated with each other across time periods [70] or structural topic models [71].

## Conclusions

We have analyzed the Web of Science core collection for the search term “ecosystem service*”, resulting in a dataset of over 14,000 articles We used a computational science method, latent Dirichlet allocation (LDA) analysis to derive main topics from the articles’ abstracts. We analyzed three periods of ES research, from 1990 to 2016 and qualitatively linked the topics between the periods in order to display research (dis-)continuities.

LDA analysis allowed us a broad and reproducible exploration into ES research content. Through a combination of LDA with qualitative interpretation of topic linkages across periods, we evaluated the question: what are the main topics in ES research and how do they evolve over time? A slight majority of topics contain natural science research on different ecosystems such as oceans, freshwater, soil, pollination or forests. Topics such as pollination, land cover, and an urban topic appear over time. Some topics are at the junction of socio-ecological land use systems (land cover, agriculture, forests, urban spaces). A smaller share of topics is based on social science approaches such as sustainable management, the role of science, valuation, and governance. This provides a counterpoint to former analyses who find a dominant share of economic and social science research in the most cited literature [3,5]. Integration across social and natural research agendas especially takes place in the area of land use topics such as agriculture, forests, conservation, and land cover. The agricultural topic is a central topic node which takes up input from the conservation topic and feeds into soil, pollination, and governance ES research.

Similar to an analysis by Gómez-Baggethun et al. [6], we indeed find a distinguishable economic valuation topic in ES research for the first two periods. Yet, in the second period we observe the occurrence of a self-reflective and critical topic on the role of science for sustainability transformations–potentially as a response to critical perspectives on a utilitarian framing. In conjunction with the valuation topic, this role of science topic feeds into both the comprehensive and transdisciplinary assessment topic [40,42], and the governance topic on institution building for integrative ecosystem (service) management [43,57].

Overall, we observe a strong natural science foundation, a growingly interdisciplinary agenda, and a notable policy-orientation of ES research. By employing a novel combination of both quantitative and qualitative content analysis methods, we contribute comprehensive evidence of an increasingly multi-faceted and integrated assessment methodology, an evolving recognition of multiple types of values in the valuation area of ES topics, and thus indications of a growing diversity of responses and instruments to meet conservation needs among ES research communities.

## Supporting information

S1 Text Readme instructions for supporting information. (DOCX)

S1 Table Top documents for topics 1990–2000. (XLS)

S2 Table Top documents for topics 2001–2010. (XLS)

S3 Table Top documents for topics 2011–2016. (XLS)

S1 Visualization LDA topic model visualization 1990–2000. (ZIP)

S2 Visualization LDA topic model visualization 2001–2010. (ZIP)

S3 Visualization LDA topic model visualization 2011–2016. (ZIP)
