# Supplementary material for: Where communities intermingle, diversity grows – The evolution of topics in ecosystem service research
Source: PLoS One. 2018 Sep 28;13(9):e0204749. doi: 10.1371/journal.pone.0204749 (PMC6161896; doi:10.1371/journal.pone.0204749)
Supplement: S1 Text — (DOCX) [file pone.0204749.s001.docx]

**The excel sheets S2-S4**

- The three “Top Documents for Topics [’STARTYEAR’_’ENDYEAR’].xls” files provide the top 20 most probable documents for each of the period’s topics, which can be found in the sheets, where sheets are labeled with topic names. Abstracts are included besides bibliometric information.

**The interactive visualizations S5-S7**

As an introductory guideline the following video may be helpful: <https://youtu.be/IksL96ls4o0>

The “LDA topic model visualization [’STARTYEAR’_’ENDYEAR’].zip” has to be downloaded and extracted to hard-drive. There is one compressed folder with interactive visualization for each period. For each period there is a topicmodelvis_[‘period’]_index.html file in the corresponding folder. The .html files should be opened with Firefox; other browsers may not perform as expected. Once opened there is a two-sided interactive visualization.

Left hand side:

- shows a bubble chart on principle component axes (that measure intertopic distances);
- bubble sizes reflect the relative share of topics in terms of assigned documents
  - if unselected, the represent overall relative share of within entire corpus of documents
  - if selected (fix selection with a click or the menu), the right-hand side shows the most relevant terms for the selected topic.

Right hand side:

- it is a bar chart showing either salient or relevant terms
  - if no topic is selected the most salient (see definition in footnote) terms are displayed.
  - if one topic is selected, the most relevant terms (see definition in footnote) are displayed.
- The slide that changes λ adjusts the computation of relevance. A value of 1 shows more frequent terms and a value of 0 shows more exclusively relevant terms for a selected topic.

Topic labels can be found below the graphs.
