# Supplementary material for: Where communities intermingle, diversity grows – The evolution of topics in ecosystem service research
Source: PLoS One. 2018 Sep 28;13(9):e0204749. doi: 10.1371/journal.pone.0204749 (PMC6161896; doi:10.1371/journal.pone.0204749)
Supplement: S1 Visualization — (ZIP) [file pone.0204749.s005.zip › topicmodelvis_1990_2000_index.html]

LDAvis


**Topic names:**
[1] 'risk mgnt' [2] 'sust. mgnt' [3] 'valuation' [4] 'global awaren.' [5] 'marine' [6] 'ecosystem fcts.' [7] 'conservation' [8] 'forests' [9] 'freshwater'
