# Supplementary material for: Where communities intermingle, diversity grows – The evolution of topics in ecosystem service research
Source: PLoS One. 2018 Sep 28;13(9):e0204749. doi: 10.1371/journal.pone.0204749 (PMC6161896; doi:10.1371/journal.pone.0204749)
Supplement: S2 Visualization — (ZIP) [file pone.0204749.s006.zip › topicmodelvis_2001_2010_index.html]

LDAvis


**Topic names:**
[1] 'role of science' [2] 'marine' [3] 'agriculture' [4] 'pollination' [5] 'land cover' [6] 'valuation' [7] 'forests' [8] 'soils' [9] 'freshwater'
