# Supplementary material for: Where communities intermingle, diversity grows – The evolution of topics in ecosystem service research
Source: PLoS One. 2018 Sep 28;13(9):e0204749. doi: 10.1371/journal.pone.0204749 (PMC6161896; doi:10.1371/journal.pone.0204749)
Supplement: S3 Visualization — (ZIP) [file pone.0204749.s007.zip › topicmodelvis_2011_2016_index.html]

LDAvis


**Topic names:**
[1] 'governance' [2] 'assessment' [3] 'land cover' [4] 'marine' [5] 'soils' [6] 'pollination' [7] 'freshwater' [8] 'forests' [9] 'urban'
